# 

**Published:** 1852-01

**Authors:** 


### CONTENTS.

PAGE.

Article I.—Mucous Membrane of the Mouth. By Professor W. R. Handy, M. D., . . . . . . . . 169

Article II.—Resolution and Argument. By E. Townsend, D. D. S., 183

Article III.—On the Preservation of the Teeth. By H. L. Jacob, Esq., M. R. C. S., &c. England, . . . . . 192

Article IV.—Report of Discussions and Proceedings of the Mississippi Valley Association of Dental Surgeons, By Allen Leslie, 208

Article V.—A Description of an Artificial Palate, Velum, Uvula, Septum of the Nose, Alveolar Process and Artificial Tooth. By Joseph Linderer, Dentist, Berlin, Prussia, . . 258

Article VI.—Mr. Gilbert and his Perpendicular Extraction, . 264

Article VII.—Report of two Cases—one of Alveolar Abscess, and the other of the injury of the Inferior Maxillary. By C. Bew, 267

Article VIII.—The Alloys of Gold for Dental Purposes. By James Robinson, . . . . . . . . . 269

Article IX.—Letter from Professor J. Allen, . . . . . 285

### SELECTED ARTICLES.

Article X.—Teeth—Comparative Anatomy, . . . . . 286

Article XI.—Case of Destruction of the Lower Jaw and of a portion of the Face. By Frank H. Hamilton, . . . . 306

Article XII.—On a case of Extensive Injury to the Jaw, following the Removal of an Incisor Tooth by the Keyed Instrument. By Thomas Underwood, Esq., . . . . . . 309

Article XIII.—On the Dissolving of Teeth in Solution of Sugar. 311

Article XIV.—Removal of a Tumor imbedded in the Parotid Gland, 312

### BIBLIOGRAPHICAL.

A Systematic Treatise. By Daniel Drake, M. D., . . . . 313

Stricture of the Urethra. By Robert Wade, F. R. C. S. &c., 320

The Teeth and their Preservation. By Charles Vasey, . . 321

The Physiology of the Teeth properly applied to their Care and Preservation. By Joseph Snake, . . . . . . 321

Practical Observations on the Teeth. By Henry Jordan, . 322

The Transactions of the American Medical Association, . . 323

Eighth Report to the Legislature of Massachusetts. By A. Walker, 324

Address Delivered before the Pennsylvania Society of Dental Surgeons. By Elisha Townsend, D. D. S., . . . . . . 325

### EDITORIAL DEPARTMENT.

Attachment of Single Teeth to a Plate, by means of a Silicious Composition, . . . . . . . . . . . 328

Philadelphia College of Dentistry, . . . . . . . 330

New York College of Dental Surgery, . . . . . . 331

Another College of Dental Surgery Contemplated, . . . 332

Gutta Percha as a Base for Artificial Teeth, . . . . . 332

Dr. G. W. Parmly, . . . . . . . . . . 333

Comparative Anatomy of the Teeth, . . . . . . 333

Dr. C. G. Davis, . . . . . . . . . . . 334

Sponge Gold, . . . . . . . . . . . 334

A New Article for Drying Cavities in Teeth, preparatory to Filling, 335

Errata, . . . . . . . . . . . . . 335

The late Mr. Durance George, . . . . . . . . 335

